# Blood Biomarkers of Neurodegeneration over Four Decades After Toxic Oil Syndrome: A Case-Control Study

**DOI:** 10.3390/ijms26115122

**Published:** 2025-05-27

**Authors:** Mariano Ruiz-Ortiz, José Lapeña-Motilva, Verónica Giménez de Bejar, Fernando Bartolomé, Carolina Alquézar, Minerva Martínez-Castillo, Sonia Wagner-Reguero, Teodoro del Ser, María Antonia Nogales, Sonia Álvarez-Sesmero, Montserrat Morales, Cecilia García-Cena, Julián Benito-León

**Affiliations:** 1 Department of Neurology, 12 de Octubre University Hospital, 28041 Madrid, Spain; mariano.ruiz.ortiz@gmail.com (M.R.-O.); jose.lapena@salud.madrid.org (J.L.-M.); neuro.gimenezdebejar@gmail.com (V.G.d.B.); 2 Group of Neurodegenerative Diseases, Hospital Universitario 12 de Octubre Research Institute (imas12), 28041 Madrid, Spain; fbartolome.imas12@h12o.es (F.B.); carolinaalquezar.imas12@h12o.es (C.A.); 3 Network Center for Biomedical Research in Neurodegenerative Diseases (CIBERNED), 28029 Madrid, Spain; 4 Alzheimer’s Centre Reina Sofía-CIEN Foundation, Instituto de Salud Carlos III, 28031 Madrid, Spain; mmartinez@fundacioncien.es (M.M.-C.); swagner@fundacioncien.es (S.W.-R.); tdelser@fundacioncien.es (T.d.S.); 5 Department of Internal Medicine, 12 de Octubre University Hospital, 28041 Madrid, Spain; mariaantonia.nogales@salud.madrid.org (M.A.N.); montserrat.morales@salud.madrid.org (M.M.); 6 Department of Psychiatry, 12 de Octubre University Hospital, 28041 Madrid, Spain; sasesmero@gmail.com; 7 ETSIDI-Center for Automation and Robotics, Universidad Politécnica de Madrid, 28012 Madrid, Spain; cecilia.garcia@upm.es; 8 Department of Medicine, Faculty of Medicine, Complutense University of Madrid, 28040 Madrid, Spain

**Keywords:** toxic oil syndrome, neurofilament light chain (NfL), glial fibrillary acidic protein (GFAP), pTau217, neurodegeneration, biomarkers, environmental toxins

## Abstract

Toxic oil syndrome (TOS) is a multisystemic disease that emerged in Spain in 1981 due to the ingestion of aniline-adulterated rapeseed oil fraudulently sold as olive oil. Although neurological sequelae, including cognitive deficits, have been documented in long-term survivors, it remains unclear whether TOS leads to chronic or progressive neurodegeneration. In this case-control study, we measured blood concentrations of neurofilament light chain (NfL), glial fibrillary acidic protein (GFAP), and phosphorylated tau 217 (pTau217) in 50 individuals with clinically confirmed TOS and 50 matched healthy controls. Biomarkers were quantified using ultrasensitive immunoassay platforms (Quanterix SIMOA SR-X and Fujirebio Lumipulse G600II). Group differences were evaluated using non-parametric tests, and multiple linear regression was applied to assess associations between biomarkers and clinical variables. While NfL levels were slightly higher in TOS patients (*p* = 0.025), no significant group differences were observed for pTau217 or GFAP. Age was a consistent predictor of biomarker levels, particularly for GFAP and pTau217, and female sex was independently associated with higher GFAP concentrations. Lower educational attainment was linked to increased NfL levels. Clinical status (TOS vs. control) did not significantly predict biomarker concentrations in any model. These findings suggest no evidence of overt or ongoing neurodegeneration in long-term TOS survivors as detected by current blood biomarkers. However, the possibility of subtle, compartmentalized, or slowly evolving neurotoxic processes cannot be excluded. Future longitudinal studies incorporating serial biomarker assessments, advanced neuroimaging, and oxidative stress markers are warranted to clarify the long-term neurological consequences of TOS and to detect subclinical trajectories of delayed neurotoxicity in this population.

## 1. Introduction

Toxic oil syndrome (TOS) is a multisystemic disorder that emerged in Spain in May 1981 following the ingestion of aniline-adulterated rapeseed oil fraudulently sold as olive oil [1]. The outbreak affected more than 20,000 individuals and led to over 300 deaths within the first year. Clinically, TOS manifested initially with respiratory symptoms, progressing to a chronic phase marked by severe myalgia, eosinophilia, peripheral neuropathy, and scleroderma-like cutaneous changes [2,3].

Although its precise pathophysiology remains incompletely understood, current evidence supports a type I hypersensitivity reaction potentially triggered by fatty acid anilides—compounds formed through the interaction of oleic acid and aniline. This mechanism is consistent with the clinical overlap observed between TOS, myalgia-eosinophilia syndrome, and scleroderma [4].

Three distinct clinical stages have been described: (1) an acute phase (~2 months) characterized by pulmonary edema, rash, eosinophilia, and myalgia; (2) an intermediate phase (months 2–4) marked by peripheral edema, skin induration, hepatic dysfunction, and pulmonary hypertension; and (3) a chronic phase (beyond month 4, with potential partial recovery after two years), featuring scleroderma-like skin changes, sicca syndrome, polyneuropathy, joint contractures, musculoskeletal pain with functional impairment, weight loss, and neuropsychiatric symptoms [4,5,6,7].

Long-term neurological consequences have been reported among TOS survivors. In a study conducted 18 years post-outbreak, exposed individuals (n = 80) demonstrated significantly greater neurological impairment compared to matched controls (n = 79), including reduced hand strength, increased vibrotactile thresholds, diminished heart rate variability (suggestive of dysautonomia), and cognitive deficits [8]. Neuropathological findings such as inflammatory neuropathy and neurogenic muscle atrophy support a neurotoxic mechanism [9], further corroborated by autopsy evidence of central chromatolysis and vacuolization in spinal motor neurons, the reticular formation, and brainstem nuclei [10]. Memory impairment remains one of the most commonly reported symptoms. A case-control study of TOS patients with subjective cognitive complaints revealed deficits in attention, episodic and semantic memory, and information processing speed—consistent with a central neurotoxic origin rather than mood-related causes [11].

Several mechanisms have been proposed to explain the neurodegenerative potential of TOS. One involves vascular and immune-mediated injury, whereby fatty acid anilides may disrupt the blood-brain barrier by promoting endothelial dysfunction and sustained proinflammatory cytokine release, increasing central nervous system vulnerability [4,12]. Another proposed mechanism is oxidative stress: these compounds can generate peroxides, triggering oxidative damage, neuroinflammation, and mitochondrial dysfunction—well-established contributors to neurodegeneration [13,14]. A third hypothesis proposes direct neurotoxicity, although the cytotoxic effects of these compounds on neural tissue remain poorly characterized. Patients with TOS and neuromuscular symptoms showed increased levels of homovanillic acid and 5-hydroxyindoleacetic acid (5-HIAA) in cerebrospinal fluid [15]. Interestingly, mice treated with oleyl anilide did not develop clinical or histological signs compatible with TOS but exhibited serotonin depletion and elevated 5-HIAA levels, suggesting that toxic agents involved in TOS induced changes in monoamine neurons of the brain [15].

Notably, these mechanisms closely resemble those implicated in classical neurodegenerative disorders [14]. Given the persistence of neuropsychiatric symptoms and the reported decline in quality of life among survivors, it is plausible that TOS may act as a trigger for a slowly progressive neurodegenerative process extending beyond the acute phase.

Over the past decade, the use of blood-based biomarkers for detecting neurodegeneration has gained increasing attention. Neurofilament light chain (NfL), measurable in both cerebrospinal fluid and plasma, has emerged as a sensitive marker of axonal injury, useful for distinguishing neurodegenerative diseases from primary psychiatric conditions [16,17,18,19]. Similarly, glial fibrillary acidic protein (GFAP), released by reactive astrocytes, has been proposed as a more specific marker of central nervous system astrogliosis and neurodegeneration [20,21], with elevated levels reported in Alzheimer’s disease (AD) [22], Parkinson’s disease [23], multiple sclerosis [24], and traumatic brain injury [25]. Moreover, plasma phosphorylated tau isoforms—especially pTau217 and pTau231—are strongly associated with amyloid positron emission tomography positivity, making them promising candidates for early detection of AD pathology [26].

Based on this rationale, the present study aims to assess blood concentrations of NfL, GFAP, and pTau217 in individuals with a history of TOS compared to matched healthy controls. Our goal was to determine whether these long-term survivors exhibit biomarker profiles suggesting chronic progressive neurodegeneration potentially attributable to the original toxic exposure.

## 2. Results

The study included 50 individuals with clinically confirmed TOS and 50 healthy controls, matched for age and sex. There were no significant differences between groups in sex distribution, mean age, or years of education, according to selection rules. The TOS group exhibited a significantly higher prevalence of arterial hypertension (66% vs. 18%; *p* < 0.001) and diabetes mellitus (24% vs. 6%; *p* = 0.025). A summary of demographic and clinical characteristics is presented in Table 1.

**Figure 1:**
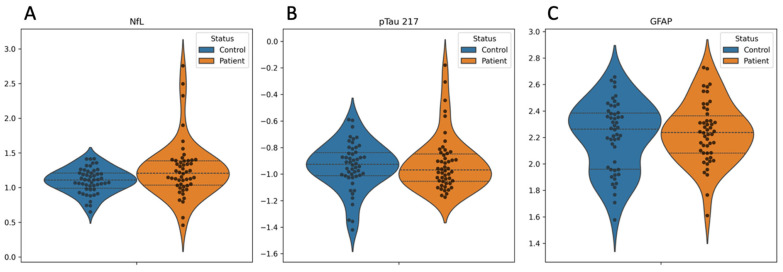
Group comparisons of blood biomarker levels in toxic oil syndrome (TOS) patients and matched controls. Violin plots show the distribution of blood concentrations of (**A**) neurofilament light chain (NfL), (**B**) phosphorylated tau 217 (pTau217), and (**C**) glial fibrillary acidic protein (GFAP) in TOS patients (orange) and matched controls (blue). Individual data points are overlaid using swarm plots. NfL and pTau217 values were log-transformed to normalize distributions. Thick dashed lines indicate group medians; thin dashed lines represent the interquartile range (IQR). NfL levels were significantly higher in TOS patients compared to controls (*p* = 0.025, Mann–Whitney U test), while no significant differences were observed for pTau217 (*p* = 0.454) or GFAP (*p* = 0.860). These plots illustrate both between-group variability and within-group distributions.

Blood biomarker concentrations are shown in Figure 1. Compared to controls, the TOS group demonstrated significantly elevated blood NfL levels (*p* = 0.025), although this difference appeared to be driven by a small number of outliers (Figure 1A). No significant group differences were observed in blood concentrations of pTau217 or GFAP (Figure 1B and Figure 1C, respectively).

**Figure 2:**
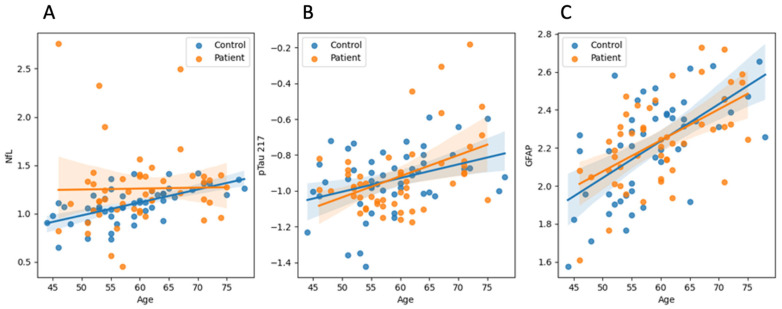
Association between age and blood biomarker concentrations in toxic oil syndrome patients and controls. Scatterplots illustrate the relationship between age and plasma levels of (**A**) neurofilament light chain (NfL), (**B**) phosphorylated tau 217 (pTau217), and (**C**) glial fibrillary acidic protein (GFAP), with toxic oil syndrome patients shown in orange and controls in blue. Linear regression lines are fitted separately for each group, with shaded areas representing the 95% confidence intervals. NfL and pTau217 values are displayed on a logarithmic scale. In pooled analyses across both groups, age showed a significant positive association with all three biomarkers, most prominently for GFAP (β = 0.018, *p* < 0.001), followed by pTau217 (β = 0.009, *p* < 0.001) and NfL (β = 0.008, *p* = 0.046). These findings are consistent with age-related increases in neurodegeneration- and glial-related markers in blood.

**Figure 3:**
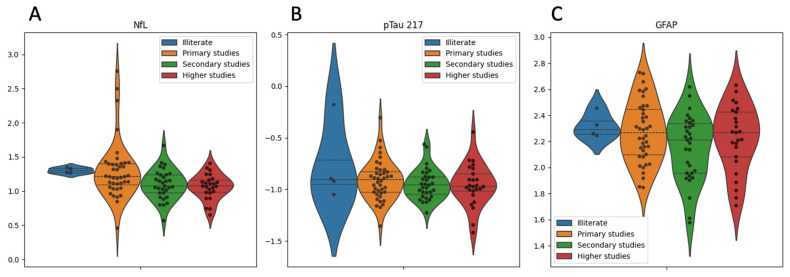
Blood biomarker concentrations by educational attainment. Violin plots show the distribution of plasma levels of (**A**) neurofilament light chain (NfL), (**B**) phosphorylated tau 217 (pTau217), and (**C**) glial fibrillary acidic protein (GFAP) across four levels of education: illiterate (blue), primary (orange), secondary (green), and higher education (red). Individual data points are displayed using swarm plots. NfL and pTau217 values were log-transformed to reduce distributional skew. Horizontal dashed lines indicate group medians and interquartile ranges. In multivariable regression analyses, educational attainment was significantly associated with lower NfL concentrations (standardized β = −0.31, *p* = 0.020), suggesting a potential link between lower education and increased neuroaxonal injury. No significant associations were observed between education and pTau217 or GFAP levels. The figures are shown with logarithmic data instead of z-scores to facilitate visualization.

We performed multiple linear regression analyses using z-scored values of GFAP, NfL, and pTau217 as dependent variables to explore predictors of biomarker concentrations. Age emerged as a robust predictor, showing significant positive associations with GFAP (*p* < 0.001), pTau217 (*p* < 0.001), and NfL (*p* = 0.046), indicating higher biomarker levels among older individuals (Figure 2). Sex was independently associated with GFAP, with significantly higher levels observed in women compared to men (*p* = 0.016). Educational attainment demonstrated a significant inverse association with NfL (*p* = 0.020), suggesting a potential protective effect of higher education (Figure 3A), although its associations with GFAP (*p* = 0.073) and pTau217 (*p* = 0.906) were not statistically significant (Figure 3B and Figure 3C, respectively). Diabetes mellitus showed a marginal association with increased pTau217 levels (*p* = 0.063), while arterial hypertension displayed a non-significant trend toward lower NfL levels (*p* = 0.071). Neither clinical status (TOS vs. control) nor the presence of peripheral neuropathy significantly predicted any biomarker levels (all *p* > 0.10).

## 3. Discussion

In this case-control study, we investigated blood concentrations of NfL, GFAP, and pTau217 in long-term TOS survivors four decades after the initial outbreak. We aimed to explore whether these blood-based biomarkers of neuroaxonal injury and neurodegeneration could reveal evidence of chronic or progressive neurological involvement in this unique population.

While NfL levels were significantly higher in the TOS group in unadjusted analyses (*p* = 0.025), this difference did not remain statistically significant after multivariate adjustment. The observed group difference appears to have been influenced by a small number of high outliers. This finding suggests that ongoing neuroaxonal degeneration is unlikely to be a predominant process in most long-term TOS survivors, while a few cases showing very high levels of plasma NfL could have experienced marked neuroaxonal damage. This stands in contrast to well-characterized neurodegenerative disorders, such as AD or frontotemporal dementia, where elevated NfL levels in blood or cerebrospinal fluid consistently reflect progressive neuronal loss [16,17,18]. Nevertheless, subtle elevations in NfL—especially in individuals with persistent symptoms—may still indicate residual effects of past toxic or inflammatory insults, and peripheral or central axonal damage cannot be entirely excluded. The overall stability of NfL levels aligns with prior clinical studies showing that the neurological manifestations of TOS were disabling but tended to plateau over time rather than follow a progressive trajectory [8].

Recent research on inherited peripheral neuropathies, particularly Charcot–Marie-Tooth disease (CMT), offers valuable insight into the longitudinal dynamics of plasma NfL. In a combined cross-sectional and longitudinal study, Rossor et al. [27] found that plasma NfL concentrations were significantly elevated in patients with CMT1B, CMT1X, and CMT2A compared to controls. However, in the cross-sectional U.S. cohort, NfL levels did not correlate with disease severity as measured by the weighted CMT Examination Score (CMTES) or the CMT Neuropathy Score (CMTNS). In contrast, the longitudinal U.K. cohort revealed a significant correlation between NfL levels and CMTES at the six-year follow-up (Spearman ρ = 0.53, *p* = 0.004), although changes in NfL over time were not associated with changes in clinical scores. Notably, NfL levels remained stable in patients with CMT1A and HSN1, while a small but significant decline was observed in those with CMT1X [27]. These findings suggest that plasma NfL is most elevated during early or active phases of axonal injury but tends to plateau or diminish in chronic or slowly progressive conditions. A comparable trajectory may apply to TOS, where a past toxic exposure could have triggered acute or subacute neuroaxonal damage, followed by a prolonged quiescent phase during which ongoing degeneration is no longer detectable through current peripheral biomarkers.

The analysis of pTau217 was designed to screen for covert AD pathology, motivated by the high prevalence of memory and other cognitive complaints in these patients [11]. Our results showed no significant differences between TOS patients and controls, providing no support for the hypothesis that AD-like tau pathology underlies the cognitive complaints observed in these individuals. These findings are consistent with previous neuropsychological and pathological studies that have implicated toxic or vascular mechanisms—rather than classic neurodegeneration—as the principal contributors to TOS-related cognitive dysfunction [9,10].

GFAP was included as an exploratory biomarker of astrocytic activation. Although no group differences were detected, GFAP levels increased with age and were higher in women, consistent with prior literature in both healthy aging and neurodegenerative contexts [21,22]. The absence of elevated GFAP in TOS patients may suggest that any acute-phase astrogliosis has already been resolved and that astroglial reactivity is currently minimal in this chronic stage of the disease. Alternatively, chronic low-grade glial activation may persist below the detection threshold of current assays.

These results should be interpreted within the clinical context of the cohort. Participants were followed in a general clinical setting and did not undergo serial cognitive testing. Although many patients reported cognitive decline, objective longitudinal measures are lacking. Consequently, the absence of biomarker abnormalities at a late single time point does not preclude the possibility of subtle or slowly evolving neurotoxic processes that remain below detectable thresholds. This is particularly relevant given the known links between environmental toxins and neurodegeneration. In Parkinson’s disease, for example, prolonged exposure to pesticides or solvents has been associated with increased risk via mechanisms such as oxidative stress, mitochondrial dysfunction, and neuroinflammation [28,29,30]. Similar mechanisms—particularly endothelial injury, blood-brain barrier disruption, and immune-mediated neural damage—have been proposed in TOS [3,4]. It is, therefore, plausible that the initial toxic exposure to TOS may have initiated insidious, long-lasting changes in the central nervous system that are not readily captured by cross-sectional blood biomarkers.

In light of the proposed role of oxidative stress in the pathophysiology of TOS [13,14], future studies should incorporate specific peripheral markers of oxidative damage. These may provide a more direct reflection of persistent redox imbalance and mitochondrial dysfunction. Multimodal biomarker panels encompassing both neurodegeneration and oxidative stress pathways may offer greater sensitivity in detecting subtle or compartmentalized central nervous system injury in this population.

It is important to recognize that blood biomarkers in neurodegeneration are still an emerging field [19]. For instance, in progressive forms of multiple sclerosis, blood NfL levels may remain normal despite clinical deterioration, while GFAP levels better reflect disease progression [31]. The temporal dynamics, cellular specificity, and sensitivity of these markers remain active areas of investigation, especially in non-classical neurodegenerative conditions such as TOS.

This study has several limitations. Its cross-sectional design prevents the assessment of temporal changes in biomarkers or symptom progression. The modest sample size may have reduced the ability to detect subtle but clinically relevant differences. The lack of neuroimaging limits the correlation of biomarker levels with structural brain changes, and participants were not followed in specialized neurology settings, which may have hindered standardized cognitive evaluations. In addition, potential confounding factors—such as comorbidities, medication use, or lifestyle variables—were not systematically accounted for.

To overcome these limitations, future studies should incorporate longitudinal designs with serial biomarker assessments to capture dynamic changes and within-subject variability. Larger, multicenter cohorts would increase statistical power, support subgroup analyses based on symptom profiles or exposure severity, and help identify predictors of long-term neurological outcomes. The inclusion of advanced neuroimaging, oxidative stress biomarkers, and longitudinal cognitive assessments would provide a more nuanced and mechanistic understanding of the enduring effects of TOS.

In summary, our findings do not support the presence of overt or progressive neurodegeneration in long-term TOS survivors, as reflected by blood concentrations of NfL, GFAP, and pTau217. However, the possibility of subtle, localized, or slowly evolving neurotoxic injury cannot be excluded, particularly in individuals with persistent neurological symptoms. These results underscore the need for follow-up studies incorporating longitudinal biomarker assessments, advanced neuroimaging, and oxidative stress markers to better characterize the potential delayed effects of environmental neurotoxins. Establishing long-term observational cohorts and integrating clinical, molecular, and imaging data will be essential to identify vulnerable subgroups and to understand the full spectrum of chronic neurological sequelae in TOS.

Moreover, the lessons learned from the TOS cohort—one of the longest-standing human models of toxic environmental exposure—may inform research in other populations exposed to neurotoxic agents, including industrial solvents, pesticides, heavy metals, or air pollutants. Establishing mechanistic links between toxin exposure, biomarker dynamics, and long-term brain health could yield broader insights into the neurological risks associated with environmental toxicants.

## 4. Materials and Methods

### 4.1. Ethics

The study was conducted in accordance with the principles of the Declaration of Helsinki. Ethical approval was obtained from the Research Ethics Committee of the 12 de Octubre University Hospital (CEIC codes: 17/035 and 23/616). All participants provided written informed consent prior to enrollment.

### 4.2. Study Design and Setting

Between April and June 2024, individuals diagnosed with TOS and healthy controls were recruited from Madrid, one of the regions most severely affected by the epidemic outbreak in 1981. This case-control study was conducted at the 12 de Octubre University Hospital in Madrid, Spain, where all interviews and blood sampling procedures were performed.

### 4.3. Participants

TOS cases had been identified according to the diagnostic criteria established in the epidemiological studies conducted during the original outbreak [32,33], and these diagnoses were reviewed at the time of enrolment in the present study. Eligible participants were those who had experienced either the acute or the chronic phase of the syndrome. The acute phase was characterized by alveolar–interstitial pulmonary infiltrates and/or pleural effusion in the presence of absolute eosinophilia greater than 500 cells/mm^3^. The chronic phase was defined by the presence of myalgia and eosinophilia and/or at least one of the following clinical features clearly attributable to TOS: scleroderma-like skin changes, peripheral neuropathy, pulmonary hypertension, or hepatopathy.

Patients were recruited from the hospital’s specialized clinical unit for TOS, the only dedicated center in Spain providing long-term follow-up and care for affected individuals. Participants were contacted consecutively until the target sample size of 50 patients was reached.

The control group consisted of 50 individuals without a history of TOS, recruited from friends and acquaintances residing in the same geographic area. Controls were frequency-matched to cases by age (±5 years), sex, and educational attainment. They shared similar demographic, sociocultural, and environmental characteristics with the patients but did not suffer any symptoms of TOS during the original outbreak.

Exclusion criteria for both groups included a diagnosis of neurodegenerative disease (e.g., AD, Parkinson’s disease), a history of stroke, chronic renal disease, chronic alcohol misuse, or any traumatic injury or clinical condition affecting the central or peripheral nervous system.

Demographic and clinical data—including age, sex, education level, medical history, cognitive complaints, and current pharmacological treatments—were collected using a standardized structured questionnaire.

### 4.4. Biomarker Quantification

- Sample Collection and Handling

Peripheral blood samples were obtained by standard venipuncture from an antecubital vein EDTA vacutainer tube. Immediately after collection, tubes were gently inverted to mix with anticoagulant and kept at 4 °C until processing. Plasma was separated within 2 h of draw by centrifugation (2000× *g* for 10 min at 4 °C) [34]. The supernatant plasma was aliquoted into polypropylene cryotubes and stored at −80 °C until analysis. These procedures ensured optimal sample stability and minimized pre-analytical variability prior to biomarker assays [34].

- GFAP Quantification (Quanterix SIMOA SR-X)

GFAP concentrations were measured using a Single Molecule Array (SIMOA) immunoassay GFAP Discovery Kit (ref: 102336, lot: 504261; Quanterix, Billerica, MA, USA) on the Quanterix SR-X ultra-sensitive detection platform. This ultra-sensitive digital immunoassay allows the detection of GFAP at femtogram-per-milliliter levels, with a sensitivity approximately 1000-fold greater than that of conventional ELISA methods [35].

Prior to analysis, plasma samples were thawed at room temperature for 1 h, mixed on a vortex for 10 s, and centrifuged at 10,000× *g* for 5 min. Assays were carried out according to the manufacturer’s instructions, including the use of kit-provided calibrators (for standard curve generation) and two quality controls in each run. The SIMOA platform offers a broad analytical measuring range; for the GFAP assay, the lower limit of detection was approximately 0.21 pg/mL (with a functional lower limit of quantification ~0.7 pg/mL), and the dynamic range extended up to ~4000 pg/mL in plasma [36]. All sample readings fell within the linear range of the assay. Intra-assay and inter-assay coefficients of variation were <15% based on kit specifications, reflecting high precision and reliability in GFAP quantification.

- pTau217 and NfL Quantification (Fujirebio Lumipulse G600II CLEIA)

Plasma phosphorylated tau at threonine 217 (pTau217) and NfL were measured using the Lumipulse G600II automated immunoassay system (Fujirebio, Tokyo, Japan). The Lumipulse G600II is a fully automated bench-top analyzer based on a chemiluminescent enzyme immunoassay (CLEIA) principle [37,38].

Each analyte was assayed with a dedicated immunoreaction cartridge Lumipulse G pTau 217 Plasma (ref: 81472, lot: D4C4129) and Lumipulse G NfL Blood kits (ref: 81215; lot: Y8B4022) (Fujirebio, Tokyo, Japan), respectively, which incorporates a two-step sandwich immunoassay. In brief, sample plasma is first incubated with magnetic particles coated with capture antibodies specific to pTau217 or NfL, followed by a reaction with an enzyme-conjugated detection antibody. After automated washing steps, a chemiluminescent substrate is added; the ensuing enzyme-driven light emission is measured by the system’s photodetector, with luminescence intensity proportional to the analyte concentration. The CLEIA technology allows rapid analyte detection (results in ~30–60 min) and a throughput of ~60 tests/hour on the G600II platform [37,38]. All steps—sample handling, incubation, washing, and signal detection—are performed automatically by the analyzer, which improves workflow efficiency and analytical consistency [37,38].

Plasma samples were thawed for 30 min at room temperature, shaken by vortex for 10 s, and centrifuged at 2000× *g* for 5 min before the analysis. All pTau217 and NfL assays were run in accordance with the manufacturer’s protocol, including the use of 5-point calibration curves and internal controls to verify assay performance. The Lumipulse platform has been extensively validated for neurodegenerative biomarkers and is noted for its high analytical sensitivity and precision. The Quality Controls were provided by the manufacturer and were measured at the beginning of each analytic session. In comparative studies, the automated Lumipulse CLEIA assays demonstrated outstanding performance in terms of assay linearity, low intra-/inter-assay variability, and excellent reproducibility [37,38]. The analytical sensitivity (functional detection limit) of the plasma pTau217 and NfL assays is in the low pg/mL range, making them well-suited for quantifying these low-abundance biomarkers in blood. The fully automated and standardized nature of the Lumipulse G600II platform minimizes operator-dependent error and ensures reliable measurement of pTau217 and NfL in all samples [37,38]. This approach yields data of comparable quality to traditional cerebrospinal fluid assays, supporting the use of plasma pTau217 and NfL as robust biomarkers of neurodegeneration in the context of TOS survivors.

### 4.5. Statistical Analysis

All statistical analyses and figure generation were performed using Python (v3.12.2) and R (v4.4.2). The following Python libraries were employed: pandas (v2.2.3) for data handling, TableOne (v0.9.1) for descriptive statistics, and statsmodels (v0.14.4) for regression analysis.

A descriptive analysis was first conducted to characterize the study population. Age differences between groups were assessed using the Student’s *t*-test. Categorical variables—including sex, educational level (illiterate, primary, secondary, higher education), diabetes mellitus, arterial hypertension, polyneuropathy, and cognitive complaints—were compared using the chi-square test or Fisher’s exact test, as appropriate. Biomarker concentrations (NfL, GFAP, and pTau217), which were non-normally distributed, were compared between groups using the Mann–Whitney U test. Finally, linear regression models were applied to assess associations between biomarker levels and demographic or clinical variables (e.g., age, sex, cognitive complaints, comorbidities), adjusting for relevant covariates.

## Figures and Tables

**Table 1:** Demographic and clinical characteristics of the study population by group (patients vs. matched controls).

Variable	Overall	Controls	Patients	*p* Value
N	100	50	50	
Sex, n (%)				0.284 ^a^
Male	32 (32.0)	19 (38.0)	13 (26.0)	
Female	68 (68.0)	31 (62.0)	37 (74.0)	
Age, mean (SD)	59.3 (8.0)	58.7 (8.2)	59.9 (7.8)	0.449 ^b^
Education, n (%)				0.161 ^a^
Illiterate	4 (4.0)	1 (2.0)	3 (6.0)	
Primary	40 (40.0)	19 (38.0)	21 (42.0)	
Secondary	31 (31.0)	13 (26.0)	18 (36.0)	
Higher education	25 (25.0)	17 (34.0)	8 (16.0)	
Diabetes mellitus, N (%)	15 (15.0)	3 (6.0)	12 (24.0)	0.025 ^a^
Arterial hypertension, N (%)	42 (42.0)	9 (18.0)	33 (66.0)	<0.001 ^a^
Polyneuropathy, N (%)	35 (35.0)	0 (0.0)	35 (70.0)	<0.001 ^a^
Cognitive complaints, N (%)	48 (48.0)	11 (22.0)	37 (74.0)	<0.001 ^a^

^a^ Chi-square test or Fisher’s exact test, as appropriate; ^b^ Student *t* test.

## Data Availability

The original contributions presented in this study are included in the article. Further inquiries can be directed to the corresponding author.

## References

[1] Tabuenca M.J. (1981). Toxic-allergic syndrome caused by ingestion of rapeseed oil denatured with aniline. Lancet.

[2] Kilbourne E.M., Posada de la Paz M., Abaitua Borda I., Diez Ruiz-Navarro M., Philen R.M., Falk H. (1991). Toxic oil syndrome: A current clinical and epidemiologic summary, including comparisons with the eosinophilia-myalgia syndrome. J. Am. Coll. Cardiol..

[3] Gelpí E., de la Paz M.P., Terracini B., Abaitua I., de la Cámara A.G., Kilbourne E.M., Lahoz C., Nemery B., Philen R.M., Soldevilla L. (2002). Centro de Investigación para el Síndrome del Aceite Tóxico. The Spanish toxic oil syndrome 20 years after its onset: A multidisciplinary review of scientific knowledge. Environ. Health Perspect..

[4] Yoshida S.H., German J.B., Fletcher M.P., Gershwin M.E. (1994). The toxic oil syndrome: A perspective on immunotoxicological mechanisms. Regul. Toxicol. Pharmacol..

[5] Alonso-Ruiz A., Calabozo M., Perez-Ruiz F., Mancebo L. (1993). Toxic oil syndrome: A long-term follow-up of a cohort of 332 patients. Medicine.

[6] del Ser Quijano T., Esteban García A., Martínez Martín P., Morales Otal M.A., Pondal Sordo M., Pérez Vergara P., Portera Sánchez A. (1986). Evolución de la afección neuromuscular en el síndrome del aceite tóxico [Course of neuromuscular involvement in the toxic oil syndrome]. Med. Clin..

[7] Pascual-Castroviejo I. (1988). A multisystemic disease caused by adulterated rapeseed oil. Brain Dev..

[8] de la Paz M.P., Philen R.M., Gerr F., Letz R., Ferrari Arroyo M.J., Vela L., Izquierdo M., Arribas C.M., Borda I.A., Ramos A. (2003). Neurologic outcomes of toxic oil syndrome patients 18 years after the epidemic. Environ. Health Perspect..

[9] Martinez-Tello F.J., Tellez I. (1991). Extracardiac vascular and neural lesions in the toxic oil syndrome. J. Am. Coll. Cardiol..

[10] Ricoy J.R., Cabello A., Rodríguez J., Téllez I. (1983). Neuropathological studies on the toxic syndrome related to adulterated rapeseed oil in Spain. Brain.

[11] del Ser T., Espasandín P., Cabetas I., Arredondo J.M. (1986). Trastornos de memoria en el síndrome del aceite tóxico (SAT) [Memory disorders in the toxic oil syndrome (TOS)]. Arch. Neurobiol..

[12] Sweeney M.D., Sagare A.P., Zlokovic B.V. (2018). Blood–brain barrier breakdown in Alzheimer disease and other neurodegenerative disorders. Nat. Rev. Neurol..

[13] Fournier E., Efthymiou M.L., Lecorsier A. (1982). Spanish adulterated oil matter: An important discovery by Spanish toxicologists—The toxicity of anilides of unsaturated fatty acids. Toxicol. Eur. Res..

[14] Martínez-Cabot A., Morató A., Commandeur J.N., Vermeulen N.P., Messeguer A. (2007). In vitro bioactivation of 3-(N-phenylamino)propane-1,2-diol by human and rat liver microsomes and recombinant P450 enzymes. Implications for toxic oil syndrome. Chem. Res. Toxicol..

[15] del Ser T., Franch O., Portera A., Muradas V., Yebenes J.G. (1986). Neurotransmitter changes in cerebrospinal fluid in the Spanish toxic oil syndrome: Human clinical findings and experimental results in mice. Neurosci. Lett..

[16] Gaetani L., Blennow K., Calabresi P., Di Filippo M., Parnetti L., Zetterberg H. (2019). Neurofilament light chain as a biomarker in neurological disorders. J. Neurol. Neurosurg. Psychiatry.

[17] Zhao Y., Xin Y., Meng S., He Z., Hu W. (2019). Neurofilament light chain protein in neurodegenerative dementia: A systematic review and network meta-analysis. Neurosci. Biobehav. Rev..

[18] Dhauria M., Mondal R., Deb S., Shome G., Chowdhury D., Sarkar S., Benito-León J. (2024). Blood-Based Biomarkers in Alzheimer’s Disease: Advancing Non-Invasive Diagnostics and Prognostics. Int. J. Mol. Sci..

[19] Eratne D., Kang M.J.Y., Lewis C., Dang C., Malpas C.B., Keem M., Grewal J., Marinov V., Coe A., Kaylor-Hughes C. (2024). Plasma and CSF neurofilament light chain distinguish neurodegenerative from primary psychiatric conditions in a clinical setting. Alzheimers Dement..

[20] Hol E.M., Pekny M. (2015). Glial fibrillary acidic protein (GFAP) and the astrocyte intermediate filament system in diseases of the central nervous system. Curr. Opin. Cell Biol..

[21] Abdelhak A., Foschi M., Abu-Rumeileh S., Yue J.K., D’Anna L., Huss A., Oeckl P., Ludolph A.C., Kuhle J., Petzold A. (2022). Blood GFAP as an emerging biomarker in brain and spinal cord disorders. Nat. Rev. Neurol..

[22] Pereira J.B., Janelidze S., Smith R., Mattsson-Carlgren N., Palmqvist S., Teunissen C.E., Zetterberg H., Stomrud E., Ashton N.J., Blennow K. (2021). Plasma GFAP is an early marker of amyloid-β but not tau pathology in Alzheimer’s disease. Brain.

[23] Pilotto A., Ashton N.J., Lupini A., Battaglio B., Zatti C., Trasciatti C., Gipponi S., Cottini E., Grossi I., Salvi A. (2024). Plasma NfL, GFAP, amyloid, and p-tau species as Prognostic biomarkers in Parkinson’s disease. J. Neurol..

[24] Ammitzbøll C., Dyrby T.B., Börnsen L., Schreiber K., Ratzer R., Romme Christensen J., Iversen P., Magyari M., Lundell H., Jensen P.E.H. (2023). NfL and GFAP in serum are associated with microstructural brain damage in progressive multiple sclerosis. Mult. Scler. Relat. Disord..

[25] Korley F.K., Jain S., Sun X., Puccio A.M., Yue J.K., Gardner R.C., Wang K.K.W., Okonkwo D.O., Yuh E.L., Mukherjee P. (2022). Prognostic value of day-of-injury plasma GFAP and UCH-L1 concentrations for predicting functional recovery after traumatic brain injury in patients from the US TRACK-TBI cohort: An observational cohort study. Lancet Neurol..

[26] Milà-Alomà M., Ashton N.J., Shekari M., Salvadó G., Ortiz-Romero P., Montoliu-Gaya L., Benedet A.L., Karikari T.K., Lantero-Rodriguez J., Vanmechelen E. (2022). Plasma p-tau231 and p-tau217 as state markers of amyloid-β pathology in preclinical Alzheimer’s disease. Nat. Med..

[27] Rossor A.M., Kapoor M., Wellington H., Spaulding E., Sleigh J.N., Burgess R.W., Laura M., Zetterberg H., Bacha A., Wu X. (2022). A longitudinal and cross-sectional study of plasma neurofilament light chain concentration in Charcot-Marie-Tooth disease. J. Peripher. Nerv. Syst..

[28] Ball N., Teo W.P., Chandra S., Chapman J. (2019). Parkinson’s Disease and the Environment. Front. Neurol..

[29] Goldman S.M. (2014). Environmental toxins and Parkinson’s disease. Annu. Rev. Pharmacol. Toxicol..

[30] Dorsey E.R., Zafar M., Lettenberger S.E., Pawlik M.E., Kinel D., Frissen M., Schneider R.B., Kieburtz K., Tanner C.M., De Miranda B.R. (2023). Trichloroethylene: An Invisible Cause of Parkinson’s Disease?. J. Parkinsons Dis..

[31] Abdelhak A., Antweiler K., Kowarik M.C., Senel M., Havla J., Zettl U.K., Kleiter I., Skripuletz T., Haarmann A., Stahmann A. (2024). Serum glial fibrillary acidic protein and disability progression in progressive multiple sclerosis. Ann. Clin. Transl. Neurol..

[32] Portera A., Franch O., Del Ser T., Battistin L., Hashim G., Lajtha A. (1983). Neuromuscular manifestations of the Toxic Oil Syndrome. Clinical and Biological Aspects of the Peripheral Nervous Diseases.

[33] Ladona M.G., Izquierdo-Martinez M., Posada de la Paz M.P., de la Torre R., Ampurdanés C., Segura J., Sanz E.J. (2001). Pharmacogenetic profile of xenobiotic enzyme metabolism in survivors of the Spanish toxic oil syndrome. Environ. Health Perspect..

[34] Arranz J., Zhu N., Rubio-Guerra S., Rodríguez-Baz Í., Ferrer R., Carmona-Iragui M., Barroeta I., Illán-Gala I., Santos-Santos M., Fortea J. (2024). Diagnostic performance of plasma pTau217, pTau181, Aβ1-42 and Aβ1-40 in the LUMIPULSE automated platform for the detection of Alzheimer disease. Alzheimers Res. Ther..

[35] Rissin D.M., Kan C.W., Campbell T.G., Howes S.C., Fournier D.R., Song L., Piech T., Patel P.P., Chang L., Rivnak A.J. (2010). Single-molecule enzyme-linked immunosorbent assay detects serum proteins at subfemtomolar concentrations. Nat. Biotechnol..

[36] Quanterix Corporation (2020). SIMOA GFAP Discovery Kit HD-1/HD-X: Data Sheet. Rev03.

[37] Arcaro M., Fenoglio C., Serpente M., Arighi A., Fumagalli G.G., Sacchi L., Floro S., D’Anca M., Sorrentino F., Visconte C. (2022). A Novel Automated Chemiluminescence Method for Detecting Cerebrospinal Fluid Amyloid-Beta 1-42 and 1-40, Total Tau and Phosphorylated-Tau: Implications for Improving Diagnostic Performance in Alzheimer’s Disease. Biomedicines.

[38] Fujirebio Europe N.V. (2025). LUMIPULSE G600II: High-Throughput Immunoassay Analyzer.

